# Dietary Pattern of Schoolgoing Adolescents in Urban Baroda, India

**DOI:** 10.3329/jhpn.v31i4.20047

**Published:** 2013-12

**Authors:** P.V. Kotecha, Sangita V. Patel, R.K. Baxi, V.S. Mazumdar, Misra Shobha, K.G. Mehta, Diwanji Mansi, Modi Ekta

**Affiliations:** ^1^Country Representative, Academy for Educational Development, A2Z, The USAID Micronutrient Project India, New Delhi 110 022; ^2^Department of Community Medicine, Baroda Medical College, Vadodara; ^3^Department of Community Medicine, GMERS Medical College, Gotri, Vadodara, India

**Keywords:** Adolescents, Dietary pattern, Nutrition, Urban, India

## Abstract

Diet plays a very important role in growth and development of adolescents, during which the development of healthy eating habits is of supreme importance. There is a dual burden of undernutrition and overnutrition in this age-group. The study assessed the food habits, food preferences, and dietary pattern of schoolgoing urban adolescents in Baroda, India. Both quantitative and qualitative methods were used in this study. A quantitative survey was carried out using a pre-tested self-administered structured questionnaire among 1,440 students from class 6 to 12 in 7 English medium and 23 Gujarati medium schools. Focus group discussions, 5 each with adolescent boys and girls, were held, along with 5 focus group discussions with teachers of Gujarati and English medium schools. Nearly 80% of adolescents had consumed regular food, like *dal*, rice, *chapati*, and vegetables, including green leafy vegetables. Nearly 50% of them had consumed chocolates, and about one-third consumed fast foods. Nearly 60% of adolescents had their breakfast daily while the remaining missed taking breakfast daily. Nearly one-third of adolescents were missing a meal once or twice a week. A large majority had consumed regular foods. However, more than half of them had consumed chocolates, soft drinks, and over one-third had taken fast foods.

## INTRODUCTION

The word ‘adolescence’ is derived from the Latin verb ‘*adolescere*’, which means “grow to maturity.” Adolescence is a grey area in the spectrum of life falling between childhood and adulthood. It is an age of transition when an individual experiences rapid growth and development, both physical and psychological and changes from being a child to an adult (1).

WHO defines adolescents as persons in the age-group of 10 to 19 years (2). In India, there are an estimated 190 million adolescents comprising over one-fifth of the entire population (3).

Adolescence is also a period when development of the reproductive system, sexual maturation, formation of identity, and gender roles set in, and issues relating to identity, gender roles, and related problems arise (4). A study conducted by Kotecha *et al.* regarding identification and ranking of problems among urban adolescents could identify problems broadly into the category of health and nutrition, academic, physical growth, and development (5).

The development of healthy eating habits is important as the rapid physical growth in adolescence is associated with increased nutritional needs. Various studies on diet and nutrition intake of adolescents and young adults in the developed world have shown that their diets are often high in fats and refined carbohydrate (6).

Adolescence is also a period of increased vulnerability to obesity. Lack of physical activity and outdoor sports, along with the consumption of fat-rich ‘junk’ foods, is the major cause of obesity among the affluent population (7).

Consumption of diet high in sugar, saturated fat, salt, and calorie content in children can lead to early development of obesity, hypertension, dyslipidaemia, and impaired glucose tolerance (8).

Some dietary patterns appear quite common among adolescents, to mention a few: snacking, usually on energy-dense foods; meal skipping, particularly breakfast, or irregular meals; wide use of fast food; and low consumption of fruits and vegetables (9-10).

Among urban adolescents in India, some of these patterns are also likely to be common but very little information is available. Therefore, this study was carried out among schoolgoing urban adolescents, with the objective to assess their food habits, food preferences, and dietary pattern in Baroda city, India.

## MATERIALS AND METHODS

A list of area-wise distribution of the schools of Baroda city was obtained from the office of the District Education Officer (DEO). The list included 177 schools affiliated to the Gujarat Secondary Education Board. Using systematic random sampling, a sample of 30 schools from this list was selected for the study. The selected sample comprised 7 English medium and 23 Gujarati medium schools, which is similar to the proportion of English and Gujarati medium schools in Baroda. The study obtained approval from the school and was started after getting ethical clearance from Institutional Review Board (IRB) of Baroda Medical College. As the list of schools was prepared based on administrative areas, the sample had proportionate representation of all the administrative wards of the city.

### Study tools

The study tools involved both quantitative and qualitative components. The study was carried out among adolescents of the selected urban schools. For quantitative component, a survey was carried out using a self-administered structured questionnaire, either in English or Gujarati, among 1,440 students who included 748 girls and 692 boys from Class 6 to 12 in 7 English medium and 23 Gujarati medium schools to include desired age-group of 10-19 years. The questionnaire was pre-tested in both the languages. The selected classes were explained the purpose of the study. Participation in the study was voluntary. Self-administered questionnaire was completed by students to ensure confidentiality; the names were omitted. The instruments were collected after checking for completeness. The questionnaire was designed to assess the dietary patterns and food habits of adolescents, in which the questions were asked on the types of foods consumed in the last 24 hours (recall method), consumption of breakfast, and their habit of skipping meals.

The quantitative data collected from this self-administered questionnaire were entered and analyzed using Epi Info (version 6.04d) software. (11) Data cleaning was carried out, checked for discrepancies, and rectified.

For qualitative component, focus group discussions (FGDs), 5 each with adolescent boys and girls were held, along with 5 focus group discussions with teachers of both Gujarati and English medium schools in either Gujarati or English language, whichever was preferred by them.

The FGDs were conducted class-wise to get an idea regarding the evolving patterns by age and note the differences. It was decided to have eight participants in each FGD. We also tried to assess their perceptions regarding healthful foods, non-healthful foods, preferences for food, their thinking about junk food, and the barriers for adopting healthful food. Researchers conducted the FGDs, and two assistants were taking notes which were then expanded on the same day but audio-recording of the same was not done because participants did not give consent for audio-recording of FGDs. FGDs were conducted in separate rooms ensuring complete privacy to the participants.

The main purpose of adding qualitative component among teachers was mainly to find out teachers’ opinion about food habits of adolescents to know the level of knowledge among the teachers about nutritional needs of adolescents, and how to improve their nutritional status. FGD transcripts were later translated into English after reviewing for accuracy. Transcripts were read several times and content analyzed using the technique of open coding. Themes were defined and identified from the text.

### Definition of variables

Fast foods were defined as foods sold in a restaurant or store which are rapidly prepared and quickly served in a packaged form for taking away while junk foods were defined as energy-dense foods with high sugar/fat/salt contents and low nutrient value in terms of protein, fibre, and vitamin and mineral contents (12).

## RESULTS

### Quantitative findings

A total of 1,440 students from 7 English medium and 23 Gujarati medium schools participated in the study. Of them, 353 students were from English medium and 1,087 from Gujarati medium. Table 1 shows the demographic profile of the study participants, of whom 52% (748) were girls, and 48% (692) were boys. Less number of students of Class 10 and 12 participated due to impending board examinations.

The age distribution of the adolescent boys and girls showed that half of them belonged to the age-group of 14-16 years. About one-third were in their early adolescence (11-13 years), and the rest belonged to the late adolescence group (17-19 years). The median age of both boys and girls was 14 years. Ninety-three percent of the respondents were Hindu. With regard to education of parents, nearly one-third of the fathers and one-fifth of the mothers were graduates, 32% of the mothers and 23% of the fathers were educated up to Class 10. Overall, educational attainment was, not surprisingly, better among fathers than among mothers.

Occupational status of their mothers showed that 75% were housewives, and 7% were professionals. Among the fathers, 13% were professionals, 24% had clerical jobs, 10% had skilled technical jobs, and 21% owned a business. The average monthly family income reported was less than Rs 5,000 in 44% of the adolescents while, in about 25%, it was between Rs 5,001 and 10,000 per month. The median family income was Rs 7,000.

Two-thirds of the adolescents came from nuclear families and the rest from joint families.

Table 2 shows the dietary pattern of these adolescents. Large majority of them (nearly 80%) had consumed regular foods, like *dal*, rice, *chapati*, and vegetables, including green leafy vegetables. Nearly half of them consumed chocolates, and about one-fourth consumed fast foods while 50% consumed bakery items

Nearly 60% of adolescents had their breakfast daily whereas 13% had breakfast only 3 to 4 days a week, and 16% had their breakfast only once or twice a week, and 12 % of adolescents never had breakfast (Table 3).

**Table 1.** Demographic details of the study population

Particulars	Total (N=1,440) (%)	
Sex	
Boys	692 (48)	
Girls	748 (52)	
Age (in completed years)	
11-13	512 (35.6)	
14-16	733 (50.9)	
17-20	195 (13.5)	
Mean	14.4 years	
Median	14 years	
Type of family	
Nuclear	936 (65)	
Joint	504 (35)	
Religion	
Hindu	1,339 (93)	
Muslim	45 (3)	
Christian	28 (2)	
Other	28 (2)	
Educational qualification (father)	Father	Mother
Non-literate	3 (0.2)	27 (1.9)
Class 7	128 (8.9)	200 (13.9)
Class 10	335 (23.2)	461 (32.0)
Class 12	226 (15.7)	171 (11.9)
Graduation	421 (29.2)	292 (20.3)
Postgraduation and higher	120 (8.3)	75 (5.2)
Others	64 (9.9)	72 (5.0)
No response	143 (4.4)	142 (9.8)
Family income	N=1097 (%)	
Less than Rs 5,000	479 (43.7)	
Rs 5,001-10,000	271 (24.7)	
Rs 10,001-15,000	108 (9.8)	
Rs 15,001-20,000	67 (6.1)	
Rs 20,001-25,000	35 (3.2)	
Rs 25,001-30,000	29 (2.6)	
Beyond Rs. 30,000	108 (9.8)	
No response	343	

In this study, nearly 55% of adolescent boys and girls had habits of taking regular meals (thrice a day and not a single meal skipped in entire week) but 30% of boys and 40% of girls missed a meal once or twice a week whereas 5% of adolescents missed their meals 3 to 4 times a week (Table 4).

**Table 2.** Various food items consumed by the adolescents in the last 24 hours

Food item	N	%
*Chapati/bhakhari*	1,242	86.3
*Rice*	1,164	80.8
*Dal*	1,091	75.8
Milk	1,081	75.1
Green leafy vegetables	1,082	75.1
Fruits and fruit juice	914	63.5
Other vegetables	828	57.5
Chocolate/pastries/sweet	811	56.3
Papads and pickles	754	52.4
Biscuits or other bakery items, like bread, toast, buns, etc.	726	50.4
Beans/legumes	664	46.1
Salads	587	40.8
*Parata/puri*	578	40.1
Cold drinks/soft drinks	564	39.2
Potato chips/namkeens/deep-fried snacks	553	38.4
Ice cream/milkshake/paneer	519	36.0
Cheese/butter	440	30.6
Pizza/burger/Frankie or any other fast food (including Chinese food)	351	24.4
Egg	317	22.0
Meat/fish/chicken	190	13.2

**Table 3.** Consumption pattern of breakfast among boys and girls

Gender	Daily		3-4 days a week		1-2 day(s) a week		Never		Total
	N	%	N	%	N	%	N	%	N
Boys	409	59.1	100	14.5	111	16.0	72	10.4	692
Girls	429	57.4	88	11.8	121	16.2	110	14.7	748
Total	838	58.2	188	13.1	232	16.1	172	12.6	1,440

**Table 4.** Pattern of missing meals among adolescents

Gender	Never		1-2 times a week		3-4 times a week		No response		Total
	N	%	N	%	N	%	N	%	N
Boys	418	60.4	194	28.0	34	4.9	46	6.6	692
Girls	380	50.8	297	39.7	34	4.5	37	4.9	748
Total	798	55.4	491	34.1	68	4.7	83	5.8	1,440

### Qualitative findings

Following themes emerged during FGDs with adolescents.

#### Healthy eating habit

When adolescents were asked about meaning of healthy eating, majority of them correctly responded that healthy eating habits meant eating green leafy vegetables, nuts, sprouted pulses (*mung, chana*), fruits, and drinking milk. However, among a few students, the knowledge regarding healthful foods was lacking.

A 14 years old girl studying in a Guajarati medium school mentioned “Eating *Pani puri* from *lari”* (street-food) as a healthful food.

#### Junk foods

When asked to give their definitions and perceptions of junk food, they did so not only in terms of content but also in terms of their consequences. Adolescents attributed the following characteristics to junk food: high in sugar, fat, and calories; bad for health, and lacking nutritional value.

Specific foods listed as junk food included: chocolates, potato chips, soft drinks, cookies, cake, brownies, pizza, ice cream, and French fries.

A boy from Class 7 in an English medium school said that he liked to eat chocolates, and he also offered the same to his peers. In his words, “I love chocolates, and I eat chocolates daily and also give (these) to my friends, and so they call me chocolate boy.”

#### Food preference

To give us a better understanding of food preferences of adolescents, almost unanimous response was “fast foods, junk food, and sweets.” When asked what adolescents tend not to eat, the majority said, “fresh or cooked green leafy vegetables and fruits.”

In our study, adolescents reported unhealthy eating patterns, and their food preferences were high in fat, salt, and sugar. If these food patterns (diets high in salt and fat and low in fruits, vegetables, and fibre) continue, these could pose a risk of chronic illnesses, especially obesity, heart attack, and probably cancer.

A 13-year old boy from English medium school mentioned, “Although my weight is 70 kg and my parents have restricted the frequency of junk foods, I love pizza, and I can't control my desire for this, and so, I still consume it frequently.”

### Barriers to improving the diet

We were interested in identifying the barriers that prevented adolescents from acting upon their knowledge and practising more healthful dietary behaviours. Time was reported as the major barrier to improving the diet. Many of the adolescents told us, “We are so busy that we don't have enough time to change the food habits due to school and tuitions.”

An important barrier mentioned by a girl of Class 9 from Guajarati medium school was expressed, thus: “I need to reach tuition at 6 o'clock in the morning everyday, and so, I have no time to take breakfast except on Sundays.”

Some students also mentioned, “Many of us have preferences and strong desire to eat junk food and lack of desire to eat healthful food, even though we know the consequences of eating the junk food.”

While conducting FGDs with teachers, we incorporated three important issues: importance of nutrition in their students (adolescents), their eating habits, and how they could improve the eating habits of adolescents. Most of the teachers mentioned correctly that phase of adolescence is associated with rapid growth and development and, therefore, balanced diet is essential and failure to do so causes anaemia (*occhu lohi*: less blood), obesity (*motapanu*: fat), and other diseases, like diarrhoea and vomiting.

One of the teachers from an English medium school reported, “Many adolescent girls are self-conscious about their looks, and so they eat food in less quantity, and we call it *Aishwaria Rai Syndrome* (named after a famous actress and Miss World: so, all girls treat her as their idol) whereas it is totally the opposite among boys; they just want to build their bodies and want to become real men, which we call *Salman Khan Syndrome* (named after a famous actor with muscular build-up: so, all boys treat him as their idol). They are not able to differentiate between being muscular or fat.”

Few of the teachers advised their students about regularity of meals, eating healthful foods, like *dal*, rice, *sabji*, *roti*, sprouted seeds, buttermilk, green leafy vegetables, and fruits, not skipping meals or breakfast, and avoiding street-foods.

## DISCUSSION

Our study showed that a good number of adolescents had a healthy dietary pattern. However, more than half of them consumed chocolates, soft drinks, and over one-third had other fast foods as well, which is an area of concern.

In Nepal, a study among school children revealed that fast foods (ready-to-eat snacks, chips, etc.) were referred by more than two-thirds of them and that advertising influenced preferences in 80% (13).

A study by Punjab Agricultural University, Ludhiana, on consumption pattern of fast foods among teenagers found that fast foods are most commonly consumed between regular meals (14).

A study on the diet and nutritional status of adolescent tribal population in nine states of India found that the mean intake of all the foodstuffs, especially the income-elastic foods, such as pulses, milk and milk products, oils and fats, and sugar and jiggery, was lower than the recommended levels of Indian Council of Medical Research (ICMR) (15).

Another study of the dietary pattern, nutrient intake, and growth of adolescent girls in urban Bangladesh noticed that a substantial proportion of girls did not consume eggs (26%), milk (35%), or dark-green leafy vegetables (20%), which concurs with our findings where girls did not consume important dietary components (16).

A study on the diet quality and nutritional status of rural adolescent girls in North India assessed that subjects followed a two-meal pattern, and their diets were monotonous and cereal-based. The mean daily intake of milk and milk products, pulses, green leafy vegetables, other vegetables, and fruits were grossly inadequate (17).

In our study, majority of the boys and girls had breakfast regularly. A point of concern, however, was that 40% girls and 30% boys were missing a meal once or twice a week, mainly because they were fasting or did not have time. Some missed their meals due to ill health, lack of appetite, or disliking the food served.

In the focus group discussions, most boys correctly mentioned good and common sources of energy (such as milk, ghee, wheat, *bajri*, rice, and pulses) and vitamins (such as green leafy vegetables, fruits, and carrots) as healthful foods. Girls could correctly point out certain cooking/food preparation practices which led to the reduction in nutritive value of the food items, for instance, excessive washing of rice grains.

Most boys and girls considered stale fast foods and uncovered foods available from outside as non-healthful foods. Some of them even spoke against consumption of fried foods, soft drinks, chocolate, tea, and coffee while others admitted getting more attracted towards fast foods than homemade foods, especially when they were out with friends.

We noticed a big gap between their knowledge and practice. Despite knowing the harmful effects of unhealthy food habits, they continued to eat junk foods and the reasons being their taste preferences and strong desire to do so. Another barrier to proper healthy food habit was lack of time due to their busy schedule.

Another study relating to dietary patterns of adolescents found that the food habits during adolescence were affected by the opportunities they had of eating with peers—away from their families (18).

Most teachers felt that adolescents paid more attention to their looks; among girls to remain slim and attractive whereas among boys to appear well-built. Due to such incorrect beliefs, they did not adopt healthy eating habits.

### Conclusions

A large number of adolescents had a healthful diet and consumed regular foods. However, more than half of them consumed chocolates, soft drinks, and over one-third consumed other fast foods as well or missed a meal once or twice a week. Most boys and girls were aware of healthful and non-healthful foods. Although they were aware of the potentially-harmful effects of fast foods, they admitted a tendency to eat such foods when they were out with their friends.

### Recommendations

Based on the findings of the present study, following recommendations emerge:

1. While the food consumption includes most of the desired items, the pattern suggests that the frequency of intake of fast foods is more than desired and needs to be curtailed.
2. Regularity of food consumption is another point that needs to be stressed as 40% missed their breakfast, and 45% missed their regular meals once or more often in a week in our study, and that was found more in girls than in boys.
3. There is a need for nutrition counselling to bridge the gap between knowledge and practice. Adolescents knew what is regular and good food but their practice showed that they did not quite follow the dietary pattern that they considered good because of the social factors on one side and less-perceived importance of the regular and quality food on the other.

## ACKNOWLEDGEMENTS

We express our sincere thanks to the Government of India and World Health Organization, India Office, for funding this study. We thank Dr. Arvind Mathur, National Program Officer, WHO, India, for providing technical guidance.
